# High-throughput gene and SNP discovery in *Eucalyptus grandis*, an uncharacterized genome

**DOI:** 10.1186/1471-2164-9-312

**Published:** 2008-06-30

**Authors:** Evandro Novaes, Derek R Drost, William G Farmerie, Georgios J Pappas, Dario Grattapaglia, Ronald R Sederoff, Matias Kirst

**Affiliations:** 1 School of Forest Resources and Conservation, University of Florida, PO Box 110410, Gainesville, USA; 2 Plant Molecular and Cellular Biology, University of Florida, Gainesville, USA; 3 Interdisiplinary Center for Biotechnology Research, University of Florida, Gainesville, USA; 4 University of Florida Genetics Institute, University of Florida, Gainesville, USA; 5 Graduate Program in Genomic Sciences and Biotechnology, Universidade Católica de Brasília, Brasília, Brazil; 6 EMBRAPA Recursos Genéticos e Biotecnologia, Empresa Brasileira de Pesquisa Agropecuária, Brasília, Brazil; 7 Department of Genetics, North Carolina State University, Raleigh, USA

## Abstract

**Background:**

Benefits from high-throughput sequencing using 454 pyrosequencing technology may be most apparent for species with high societal or economic value but few genomic resources. Rapid means of gene sequence and SNP discovery using this novel sequencing technology provide a set of baseline tools for genome-level research. However, it is questionable how effective the sequencing of large numbers of short reads for species with essentially no prior gene sequence information will support contig assemblies and sequence annotation.

**Results:**

With the purpose of generating the first broad survey of gene sequences in *Eucalyptus grandis*, the most widely planted hardwood tree species, we used 454 technology to sequence and assemble 148 Mbp of expressed sequences (EST). EST sequences were generated from a normalized cDNA pool comprised of multiple tissues and genotypes, promoting discovery of homologues to almost half of *Arabidopsis* genes, and a comprehensive survey of allelic variation in the transcriptome. By aligning the sequencing reads from multiple genotypes we detected 23,742 SNPs, 83% of which were validated in a sample. Genome-wide nucleotide diversity was estimated for 2,392 contigs using a modified theta (θ) parameter, adapted for measuring genetic diversity from polymorphisms detected by randomly sequencing a multi-genotype cDNA pool. Diversity estimates in non-synonymous nucleotides were on average 4x smaller than in synonymous, suggesting purifying selection. Non-synonymous to synonymous substitutions (Ka/Ks) among 2,001 contigs averaged 0.30 and was skewed to the right, further supporting that most genes are under purifying selection. Comparison of these estimates among contigs identified major functional classes of genes under purifying and diversifying selection in agreement with previous researches.

**Conclusion:**

In providing an abundance of foundational transcript sequences where limited prior genomic information existed, this work created part of the foundation for the annotation of the *E. grandis *genome that is being sequenced by the US Department of Energy. In addition we demonstrated that SNPs sampled in large-scale with 454 pyrosequencing can be used to detect evolutionary signatures among genes, providing one of the first genome-wide assessments of nucleotide diversity and Ka/Ks for a non-model plant species.

## Background

The high-throughput and cost-effectiveness of DNA pyrosequencing using 454 Life Sciences technology [1] has been successfully applied to large-scale EST sequencing in maize [2,3], *Medicago *[4] and *Arabidopsis *[5,6], resulting in a significant contribution of additional ESTs for these species. However, these studies were carried out on organisms with extensive transcriptome sequences already available. Pre-existing sequences support the assembly of 454 reads into contigs, thereby minimizing the drawback of short average read length (100–200 bp) produced by the pyrosequencing technology. However, the benefits from recent improvements in sequencing technologies may be even more valuable for plant species with high economic value but limited genomic resources. Yet, it has not been shown whether the primary limitations of the pyrosequencing method – short read lengths and ambiguous homopolymer reads – can be overcome to produce useful information for species with essentially no prior gene sequence information.

The 454 sequencing also provides an opportunity to identify allelic variants by sequencing and aligning ESTs from several haplotypes. Recently, two 454 cDNA sequencing runs, each interrogating a single maize inbred line, were used to identify over 36,000 putative single nucleotide polymorphisms (SNPs) [7]. SNP discovery with 454 technology could also be accomplished by simultaneously sequencing multiple genotypes to sample the nucleotide diversity of an organism [8,9]. However, the assembly of individual haplotypes is not feasible when sequencing a cDNA pool from highly heterozygous individuals. The unavailability of haplotype sequences hinders the use of most statistics to calculate genetic diversity. Therefore, new models are needed to estimate population genetic parameters.

Here we describe sequencing, assembly, and SNP discovery from 454-derived EST sequences of *Eucalyptus grandis*, a species with virtually no public genome sequence information. *Eucalyptus *is the most widely planted woody angiosperm in the world [10]. Because of its value as a renewable source of raw material for wood, paper products, and biofuels, *E. grandis *has been selected for sequencing by the U.S. Department of Energy/Joint Genome Institute for completion in 2010 [11]. Accurate annotation of the *E. grandis *genome sequence will rely heavily upon a repository of expressed sequences, as was demonstrated during the annotation of the *Arabidopsis *[12] and *Populus *[13] genomes. However, only 1,939 *E. grandis *expressed sequence tags (ESTs) are currently deposited in the National Center for Biotechnology Information (NCBI) database. Thus, rapid development of a large EST sequence collection will be crucial to support the *E. grandis *genome sequence annotation and for continued advancement of *Eucalyptus *genomics research.

With the purpose of generating the first broad survey of genes in a *Eucalyptus *species, we sequenced and assembled 148 Mbp of *E. grandis *ESTs from two GS-20 and one GS-FLX 454 pyrosequencing runs. Assembled sequences (25.4 Mbp) were deposited in the GenBank representing a 37× enrichment in publicly available expressed sequences for the species. Sequences were generated from a normalized cDNA pool comprised of multiple tissues and genotypes, promoting extensive gene discovery and a comprehensive survey of allelic variation in the transcriptome. We demonstrate that 454 reads can be reassembled into contigs without utilizing traditional cDNA sequences as scaffolds. In addition, we show that SNPs detected from pyrosequencing a pool of genotypes are useful to reveal selection signatures among genes. Pyrosequencing technology can rapidly provide a foundational public genomic resource for an economically important but previously uncharacterized species.

## Results

### 454 pyrosequencing and assembly of *E. grandis *ESTs

An *E. grandis *normalized cDNA library was synthesized from RNA of vegetative tissues sampled from 21 different genotypes. Two GS-20 and one GS-FLX 454 pyrosequencing runs performed on the normalized cDNA pool generated 1,024,251 reads comprising 148.4 Mbp of sequence (Table 1). The GS-FLX platform produced more reads than the GS-20 and an average read length 2× longer. To compare the length distributions of contiguous sequences (contigs) using the different 454 platforms, we separately assembled the reads from the two GS-20 runs, the GS-FLX run alone, and from all the three sequencing runs combined (Table 2). The two GS-20 runs generated contigs measuring 131 bp on average, with 95% of the contigs being shorter than 250 bp. Despite generating only 25% more sequence than the GS-20 runs combined, the longer reads obtained with the GS-FLX platform render contigs that average almost three times longer (353 bp). The GS-FLX run allowed assembly of the *E. grandis *454-derived expressed sequences into long contigs that could be more accurately annotated. Assembly of all three pyrosequencing runs generated 71,384 contigs with an average length of 247 bp, of which 5,838 (8%) measure more than 500 bp. The addition of GS-20 reads increased the number of short contigs in the full assembly, resulting in shorter average contig length when compared to the assembly with GS-FLX reads only.

**Table 1:** Summary of *E. grandis *cDNA sequences.

**Run number**	**Method**	**Platform**	**Number of reads**	**Average length**	**Total bp**
1	454 pyrosequencing	GS-20	328,486	106.28 bp	34.9 Mbp
2	454 pyrosequencing	GS-20	303,149	102.54 bp	31.1 Mbp
3	454 pyrosequencing	GS-FLX	392,616	209.89 bp	82.4 Mbp
454 all (3 runs)	454 pyrosequencing	GS-20 + FLX	1,024,251	145.24 bp	148.4 Mbp

Sanger (control)	dideoxy sequencing	ABI 3100	86,328	522.18 bp	45.1 Mbp

Summary of the *E. grandis *expressed sequences generated with GS-20 and GS-FLX pyrosequencing runs, and control ESTs obtained from dideoxy-based sequencing of an analogous *Eucalyptus *library

**Table 2:** Summary and distribution of assembled sequences. Length distribution and characteristics of contigs assembled from the two GS-20 runs, one GS-FLX run, the three 454 runs combined and from the control Sanger sequenced ESTs

	**GS-20**	**GS-FLX**	**454 all**	**Sanger (control)**
Run(s) in assembly	1 + 2	3	1 + 2 + 3	-

≤ 100 bp	18987 (29%)	52 (<1%)	10820 (15%)	0
101 – 250 bp	42320 (66%)	17348 (62%)	35958 (50%)	0
251 – 500 bp	2476 (4%)	5355 (19%)	18768 (26%)	7564 (35%)
501 – 750 bp	535 (<1%)	2463 (9%)	2869 (4%)	9226 (43%)
751 – 1000 bp	167 (<1%)	1314 (5%)	1396 (2%)	2830 (13%)
> 1000 bp	99 (<1%)	1547 (6%)	1573 (2%)	1812 (8%)

Total contigs	64584 (100%)	28079 (100%)	71384 (100%)	21432 (100%)
Average contig length (bp)	130.6	353.22	247.16	623.35
Reads in contigs	80.72%	71.36%	88.41%	84,88%
Average reads/contig	7.89	9.98	12.69	3.42

Next we compared the sequences from 454 with 86,328 ESTs obtained from a proprietary database derived from sequencing a broad set of *Eucalyptus *cDNA libraries using conventional (Sanger) dideoxy-based sequencing (Table 1). The comparison shows that although Sanger contigs were built from a dataset with only one third the total basepairs of that derived from 454, they are on average more than twice as long (Table 2). However, the larger number of reads generated by the 454 technology substantially improves the efficiency of gene discovery, as demonstrated below.

### Annotation of the *E. grandis *ESTs

**Figure 1:**
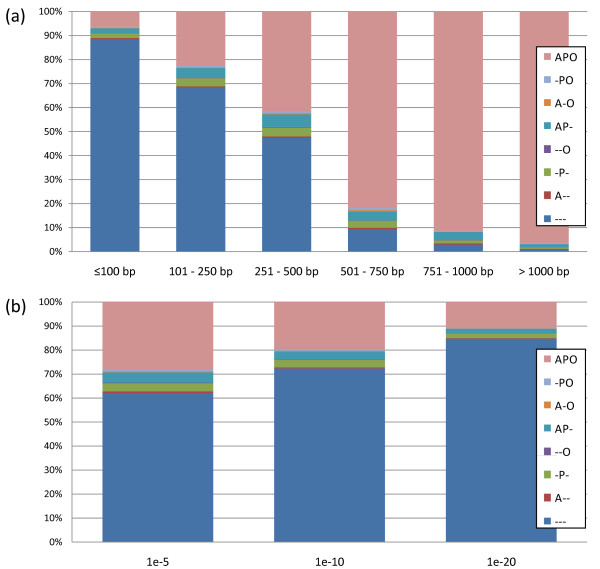
**Proportion of *E. grandis *unigenes with homology to gene models**. Proportion of *E. grandis *unigenes (contigs + singlets) without (-) and with homology to the *Arabidopsis *(A), *Populus *(P), *Oryza *(O) gene models. **(a) **Effect of the sequence length on the proportion of homology to gene models (*E *value 10^-5^). **(b) **Proportion of *E. grandis *unigenes longer than 100 bp with and without homology to gene models at three different *E *values (10^-5^, 10^-10 ^and 10^-20^).

An *E. grandis *unigene set was generated by combining all 71,384 assembled contigs and 118,722 non-assembled reads (singlets) generated by the three 454 runs. The unigene set was annotated by searching for sequence similarities using BlastX against *Arabidopsis *(TAIR v. 7.0), *Populus *(JGI v. 1.1) and *Oryza *(TIGR v. 5.0) gene models. As expected, the likelihood of finding similarity to previously described gene models is highly dependent on the length of the query sequence (Figure 1a). Logistic regression testing the effect of sequence length on whether or not the query sequence have at least one BlastX hit (*E *value 10^-5^) was highly significant (p-value < 0.00001). For instance, sequences longer than 1000 bp have significant similarity (*E *value 10^-5^) with gene models from all three species in 96% of cases, whereas 88% of sequences shorter than 100 bp have no similarities to any annotated gene model. Among 118,013 unigenes longer than 100 bp 38% have similarity to at least one gene model at an *E *value of 10^-5^, 28% at an *E *value of 10^-10^, and 15% at an *E *value of 10^-20 ^(Figure 1b). The low proportion of BlastX hits is mainly due to the high frequency of shorter sequences (75^th ^percentile = 252 bp).

**Figure 2:**
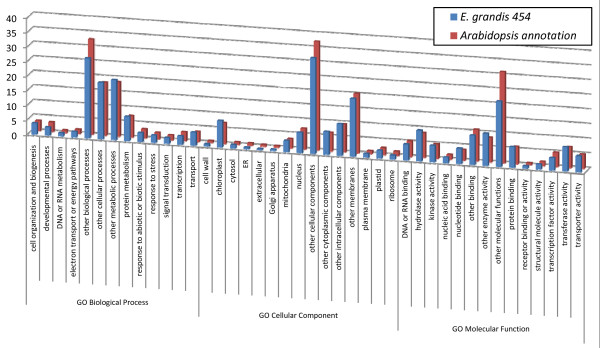
**Proportion of GO categories found in the *E. grandis *unigenes**. Proportion of categories of each Gene Ontology (GO) sampled by the *E. grandis *unigene sequences compared with the proportions found in the *Arabidopsis *genome annotation.

Next, we determined the proportion of annotated gene models for which homology was detected among *E. grandis *unigenes measuring over 100 bp. Homology was detected to 45% of *Arabidopsis*, 39% of *Populus*, and 22% of *Oryza *gene models (*E *value 10^-5^). The higher proportion of *Arabidopsis *genes that are apparent homologues to *Eucalyptus *is expected as the two species are more phylogenetically related than to *Populus *or *Oryza *[14]. *Arabidopsis *gene models are also more refined than those from the other plant species. Analyzing only the 22,032 *Arabidopsis *gene models for which there is any detected transcript evidence (TAIR v. 7.0) leads to a higher proportion of homologies: 58% with *E *value 10^-5 ^and 39% with *E *value 10^-20 ^(Table 3). Finally, we utilized the Gene Ontology (GO) classifications from the *Arabidopsis *best-hit gene models (*E *value 10^-5^). Proportions of best hits in each GO category were generally similar to those found in the *Arabidopsis *genome annotation (Figure 2). The GO annotation analysis reinforces the inference that a broad diversity of genes was sampled in our multi-tissue normalized cDNA pool.

**Table 3:** Proportion of gene models with homologies to *E. grandis *cDNAs.

**Organism**	**BlastX threshold**	**Matches against 454 unigenes**	**Matches against Sanger unigenes**
*Arabidopsis *(31,921)	1e-5	14250 (45%)	10154 (32%)
	1e-10	12347 (39%)	9561 (30%)
	1e-20	9077 (28%)	8410 (26%)
*Arabidopsis *with transcript evidence (22,032)	1e-5	12790 (58%)	9542 (43%)
	1e-10	11265 (51%)	9029 (41%)
	1e-20	8489 (39%)	8003 (36%)
*Populus *(45,555)	1e-5	17724 (39%)	11580 (25%)
	1e-10	15383 (34%)	10962 (24%)
	1e-20	11190 (25%)	9701 (21%)
*Oryza *(66,710)	1e-5	14510 (22%)	9893 (15%)
	1e-10	12139 (18%)	9193 (14%)
	1e-20	8393 (13%)	7834 (12%)

Number and percentage of gene models with matches against the *E. grandis *454 unigenes and against the control Sanger sequenced *Eucalyptus *unigenes using three different BlastX thresholds. The number of genes in each organism is presented in parenthesis in the first column.

### Efficiency of gene discovery: 454 and Sanger sequencing comparison

To compare the efficiency of gene discovery in the two sequencing platforms (454 and Sanger) we established a unigene set for the Sanger sequenced ESTs by combining the 21,432 contigs with 17,203 singlets. The Sanger unigene set has a total of 22.05 Mbp, and is therefore comparable to the 25.42 Mbp in the *E. grandis *454 unigenes measuring over 100 bp. We first aligned the two unigene sets to each other using BlastN (*E *value 10^-5^), and detected 84% of the Sanger unigenes having a match to the 454 dataset but only 41% of the 454 unigenes having homology to the Sanger sequences (data not shown). This is an indication of a greater coverage of distinct cDNAs in the 454 derived sequences. However, it is possible that the higher frequency of shorter sequences found in the 454 dataset contributed to an increased number of no-hits, as shorter sequences are less likely to align with a significant *E*-value. To rule out this possibility, Sanger unigenes were also used in an analogous BlastX against the *Arabidopsis*, *Populus *and *Oryza *gene models. For all organisms and all Blast thresholds applied, a larger number of gene models had similarity to the 454 unigenes than those generated by Sanger sequencing (Table 3). Therefore, the large number of reads generated by 454 pyrosequencing appears to maximize gene discovery in EST sequencing projects. On the other hand, mean Blast alignment lengths using the 454 unigenes are approximately 2× shorter than for the Sanger unigene set (data not shown). Thus, although the 454 unigene set samples a broader diversity of transcriptional units, this occurs at the cost of decreased length of sequence of the individual genes.

### SNP detection and validation in the *E. grandis *454-generated ESTs

The GS Reference Mapper (454 Life Science) software was used to identify polymorphisms among ESTs by aligning individual reads against contigs from the assembly. For a sequence difference to be declared a true polymorphism, at least two individual reads aligning to the consensus must have the variant allele and at least two others must have the allele of the consensus. By applying this criterion, 30,108 variants were detected in 10,223 contigs (Table 4). We analyzed only single nucleotide polymorphisms (SNPs) and excluded all 821 indels and 635 variants involving more than one nucleotide. Also, we considered only 23,742 higher confidence SNPs for which both alleles were present in at least 10% of the reads aligned at the polymorphic locus. Although this requirement reduces the sensitivity in detecting rare SNPs, it increases the specificity of true SNP detection by lowering the likelihood of including false variants that arise due to sequencing errors. Among both high and low confidence SNPs, the proportion of transition nucleotide substitutions (75 and 85%) was greater than the proportion of transversions (25 and 15%).

**Table 4:** Polymorphisms detected among *E. grandis *cDNA sequences.

**Variant type**	**Number of variants**	**Number of contigs containing SNPs**
Indel	821	704
Involving two or more nucleotides	635	537
Total SNPs	28,652	9,942
Lower confidence SNPs (freq rare allele <10%)	4,910	1,089
Transition	4,239	1,005
Transversion	671	405
Higher confidence SNPs (freq rare allele ≥ 10%)	23,742	9,845
Transition	17,871	8,394
Transversion	5,871	3,881

TOTAL	30,108	10,223

Number of detected polymorphisms and affected contigs by variant type. The higher confidence SNPs were selected for further analysis

To validate SNPs detected by GS Reference Mapper, we PCR amplified a sample of 43 contig sequences from the normalized cDNA used in the 454 sequencing. Each amplicon was sequenced bidirectionally (forward and reverse) using standard dideoxy-based sequencing in an ABI3730. Sequencing chromatograms were analyzed with Chromas 2.32 (Technelysium Pty. Ltd.), and SNPs were identified as overlapping nucleotide peaks. The number of putative SNP loci encompassed in the sequences of each amplicon ranged from 3 to 15 (Table 5). Of 337 SNP loci predicted to reside in the amplified sequences, 279 (82.8%) were validated.

**Table 5:** Validation of SNPs with conventional sequencing.

	Amplified contig	Non-validated	Validated	Number of predicted SNPs
amplicon01	KIRST.1015.C2	4 (44%)	5 (56%)	9
amplicon02	KIRST.2351.C2	4 (44%)	5 (56%)	9
amplicon03	KIRST.1461.C1	2 (40%)	3 (60%)	5
amplicon04	KIRST.1992.C1	3 (38%)	5 (63%)	8
amplicon05	KIRST.25.C1	4 (36%)	7 (64%)	11
amplicon06	KIRST.1936.C1	3 (33%)	6 (67%)	9
amplicon07	KIRST.12521.C1	2 (33%)	4 (67%)	6
amplicon08	KIRST.15421.C1	2 (33%)	4 (67%)	6
amplicon09	KIRST.2632.C1	1 (33%)	2 (67%)	3
amplicon10	KIRST.4036.C2	4 (33%)	8 (67%)	12
amplicon11	KIRST.5530.C1	2 (33%)	4 (67%)	6
amplicon12	KIRST.3079.C1	2 (29%)	5 (71%)	7
amplicon13	KIRST.15389.C1	1 (25%)	3 (75%)	4
amplicon14	KIRST.486.C3	2 (25%)	6 (75%)	8
amplicon15	KIRST.823.C1	2 (22%)	7 (78%)	9
amplicon16	KIRST.854.C4	2 (20%)	8 (80%)	10
amplicon17	KIRST.2687.C1	1 (20%)	4 (80%)	5
amplicon18	KIRST.4822.C1	1 (20%)	4 (80%)	5
amplicon19	KIRST.340.C6	2 (18%)	9 (82%)	11
amplicon20	KIRST.11157.C1	1 (17%)	5 (83%)	6
amplicon21	KIRST.152.C3	1 (17%)	5 (83%)	6
amplicon22	KIRST.8182.C6	1 (17%)	5 (83%)	6
amplicon23	KIRST.1003.C1	1 (14%)	6 (86%)	7
amplicon24	KIRST.1268.C4	1 (14%)	6 (86%)	7
amplicon25	KIRST.1975.C1	1 (14%)	6 (86%)	7
amplicon26	KIRST.4785.C1	1 (14%)	6 (86%)	7
amplicon27	KIRST.52.C8	1 (14%)	6 (86%)	7
amplicon28	KIRST.340.C1	2 (13%)	13 (87%)	15
amplicon29	KIRST.17053.C1	1 (13%)	7 (88%)	8
amplicon30	KIRST.52.C1	1 (13%)	7 (88%)	8
amplicon31	KIRST.8655.C1	1 (8%)	11 (92%)	12
amplicon32	KIRST.1285.C3	1 (7%)	14 (93%)	15
amplicon33	KIRST.1441.C5	0 (0%)	5 (100%)	5
amplicon34	KIRST.17202.C1	0 (0%)	8 (100%)	8
amplicon35	KIRST.2273.C1	0 (0%)	7 (100%)	7
amplicon36	KIRST.2790.C1	0 (0%)	11 (100%)	11
amplicon37	KIRST.2900.C3	0 (0%)	4 (100%)	4
amplicon38	KIRST.34.C15	0 (0%)	8 (100%)	8
amplicon39	KIRST.344.C1	0 (0%)	7 (100%)	7
amplicon40	KIRST.4650.C2	0 (0%)	7 (100%)	7
amplicon41	KIRST.5060.C2	0 (0%)	10 (100%)	10
amplicon42	KIRST.5120.C1	0 (0%)	10 (100%)	10
amplicon43	KIRST.6233.C1	0 (0%)	6 (100%)	6

Total	43 contigs	58 (17%)	279 (83%)	337

Number and percentage of non-validated and validated SNPs for each of the 43 amplicons sequenced with dideoxy-based method

### Analysis of synonymous and non-synonymous SNPs in *E. grandis *genes

The proportion of mutations that change amino acid sequence (i.e. non-synonymous) relative to those that do not (i.e. synonymous) can indicate whether a gene is under purifying, neutral or diversifying selection [15]. The large number of ESTs sequenced from a mixture of genotypes could provide information for a genome-wide analysis of gene evolution based on the ratio of non-synonymous (*K*a) to synonymous substitutions (*K*s). To carry out this analysis, we initially determined whether SNPs introduce synonymous or non-synonymous mutations by (1) defining the sequence reading frame with a BlastX alignment against *Arabidopsis *peptides, (2) isolating codons containing SNPs, and (3) comparing the translated amino acids for each allele. Next, the degeneracy of nucleotides in the contigs' coding sequence was evaluated to estimate non-synonymous to synonymous substitution rate (Ka/Ks). We estimated Ka/Ks for 2,001 *E. grandis *contigs that have at least three high confidence SNPs and one positive BlastX hit against *Arabidopsis *gene models (*E *value 10^-5^) [see Additional file 1]. Distribution of Ka/Ks among these contigs was right-skewed, with estimates ranging from 0.008 to 2.101 and averaging 0.30, suggesting that the majority of the genes are under purifying selection.

Gene ontology (GO) categories associated with contig annotations derived by homologies to *A. thaliana *gene models allowed us to compare frequency of GO class representation in the extremes of the Ka/Ks distribution. Two binary variables ("purifying" and "diversifying") were created to classify contigs according to their Ka/Ks – contigs with Ka/Ks smaller than 0.15 (643 contigs) were classified as "purifying", while those with Ka/Ks greater than 0.5 (273 contigs) were defined as "diversifying". Ka/Ks > 1.0 may be an overly stringent criterion of positive selection when estimated for the whole coding sequence [16], and therefore we utilized a lower threshold (0.5) for this analysis. A Fisher's exact test for each binary category was used to statistically define GO Biological Process classes enriched in the Ka/Ks distribution extremes. Table 6 depicts GO classes enriched (p-value < 0.05) among contigs on the two Ka/Ks extremes. Genes encoding proteins of the ribosome complex and involved in translation are the most significantly enriched (p-value = 0.0006) within the "purifying" classification. In addition, genes encoding proteins involved in nucleosome assembly, chromosome organization and proteosome complex also appear to be constrained by purifying selection. Among GO categories of genes undergoing diversifying selection, there is enrichment for only those with unknown function and organismal development.

**Table 6:** GO categories enriched for *E. grandis *genes under purifying and diversifying selection.

**Ka/Ks extreme^a^**	**GO category**	**Proportion out extreme**	**Proportion in extreme**	**p-value**
"purifying"	translation	0.0400	0.0769	0.0006
"purifying"	ubiquitin-dependent protein catabolic process	0.0092	0.0280	0.0023
"purifying"	nucleosome assembly	0.0008	0.0098	0.0039
"purifying"	chromosome organization and biogenesis	0.0000	0.0070	0.0056
"purifying"	ribosome biogenesis and assembly	0.0108	0.0252	0.0158
"purifying"	response to hydrogen peroxide	0.0031	0.0126	0.0169
"purifying"	response to high light intensity	0.0031	0.0112	0.0326
"diversifying"	biological_process_unknown	0.1703	0.2751	0.0002
"diversifying"	multicellular organismal development	0.0028	0.0175	0.0129

^a ^The "purifying" extreme is composed of contigs with Ka/Ks smaller than 0.15, while the "diversifying" extreme has contigs with Ka/Ks greater than 0.50.

Biological process GO categories enriched (p-value < 0.05) in each of the two extremes of Ka/Ks distribution.

### Nucleotide diversity among the *E. grandis *genes

In addition to the ratio of non-synonymous to synonymous substitutions (Ka/Ks), a measurement of nucleotide diversity can also reveal differences in selection pressure acting on different genes. However, since our sequencing method generated ESTs from a pool of 21 *E. grandis *genotypes, identification of the genotypic origin for each read becomes impossible. As a result the assembly of individual haplotypes is unfeasible and exact estimation of standard nucleotide polymorphism parameters such as theta (θ) [17] could not be obtained. Thus, we developed a relative measurement of nucleotide diversity, referred hereafter as beta (β), which normalizes the number of polymorphisms detected relative to the sequence length and depth of each contig. Sequence length and depth are variables affecting the likelihood of finding SNP when it is present in a given contig. The parameter β is estimated by the equation:

$$\beta =\frac{\left(\frac{S+1}{L}\right)}{\sum_{i=1}^{D-1}\left(1/i\right)}$$

where *S *is the number of SNPs detected in the contig, *L *is the contig sequence length and *D *is the sequencing depth estimated by the average number of reads aligned to each nucleotide position during assembly of the contig. The constant 1 was added to the number of SNPs in the numerator to enable comparisons with contigs containing no detected SNP. Contigs with extensive length and depth but for which no SNP is detected represent highly conserved genes.

There is one important distinction between β and the traditional nucleotide polymorphism estimate θ. When calculating θ, the average sequencing depth (D) variable in β is, instead, the number of different sampled haplotypes. These two variables are different because sequence reads from 454 represent a random sample from the pool of haplotypes in the cDNA library. Therefore some haplotypes may be sequenced multiple times, while others may not be sampled at all. Although this complicates comparisons with θ estimates from other studies, β is useful as a relative measurement to compare the nucleotide diversity between contigs generated within this project.

We estimated β for the 2,392 contigs with coding sequence measuring more than 200 bp and an average sequencing depth of at least 10 reads/nt [see Additional file 2]. These thresholds reduce imprecision in the proportion of SNPs per nucleotide as well as the likelihood that all reads aligned to the contig on a given nucleotide arise from the same haplotype. Three β parameters were calculated for each contig: β_T_, which estimates the diversity on the entire contigs, including its non-coding regions; β_N_, which estimates the diversity in non-synonymous sites; and β_S_, which estimates the diversity in synonymous sites. As observed for the Ka/Ks ratio, the distributions of all three nucleotide diversity parameters (Table 7) were right-skewed, suggesting that the majority of *E. grandis *transcribed regions are constrained by purifying selection. Average values of β_S _were more than 4× larger compared with diversity in non-synonymous nucleotides (β_N_), offering further evidence for the action of natural selection on the genetic diversity detected in these genes.

**Table 7:** Summary of diversity in expressed sequences of *E. grandis*.

**Parameter**	**Mean**	**Median**	**Range**
β_T_	1.86 × 10^-3^	1.65 × 10^-3^	1.22 × 10^-4 ^– 9.11 × 10^-3^
β_N_	1.81 × 10^-3^	1.49 × 10^-3^	1.35 × 10^-4 ^– 15.14 × 10^-3^
β_S_	7.88 × 10^-3^	6.19 × 10^-3^	6.67 × 10^-4 ^– 48.15 × 10^-3^

Distribution summary of three nucleotide diversity parameters estimated for 2,392 contigs.

The annotation of each contig from homology to *A. thaliana *gene models was again used to compare GO categories enriched in the extremes of the β_N _distribution. Similarly to Ka/Ks, two binary variables were empirically created to identify genes on the "conserved" and "diverse" extremes of the β_N _distribution. The "conserved" classification has 605 contigs with β_N _smaller than 1.0 × 10^-3^. The "diverse" classification has 209 contigs with β_N _greater than 3.5 × 10^-3^. Fisher's exact test for each binary category detected GO classes enriched among each of the two β_N _extremes (Table 8). As suggested by the Ka/Ks analysis for genes undergoing purifying selection, the "conserved" extreme of β_N _is also enriched for genes encoding proteosome core factors involved in protein degradation. In addition, in the conserved extreme we also found enrichment for genes involved in malate metabolism. Among contigs classified as "diverse", there is enrichment for genes of unknown function and for genes involved in defense response, including response to biotic stimuli.

**Table 8:** GO categories enriched among conserved and diverse genes of *E. grandis*.

**β_N _extreme^a^**	**GO category**	**Proportion out extreme**	**Proportion in extreme**	**p-value**
"conserved"	malate metabolic process	0.0007	0.0093	0.0056
"conserved"	ubiquitin-dependent protein catabolic process	0.0131	0.0278	0.0314
"diverse"	defense response	0.0070	0.0302	0.0069
"diverse"	biological_process_unknown	0.1647	0.2362	0.0134
"diverse"	response to biotic stimulus	0.0032	0.0151	0.0480

^a ^The "conserved" tail is composed of contigs with β_N _smaller than 1.0 × 10^-3^, while the "diverse" tail has contigs with β_N _greater than 3.5 × 10^-3^.

Biological process GO categories enriched (p-value < 0.05) in each of the two extremes of β_N _(non-synonymous nucleotide diversity) distribution.

## Discussion

Our analysis of 148.4 Mbp of *E. grandis *expressed sequences generated with three 454 sequencing runs demonstrates that short reads produced with pyrosequencing technology can be assembled *de novo *into reasonably long contigs; an advantage for species with limited public genomic resources. The 25.4 Mbp comprised in our unigene set represents an enrichment of 37× in the amount of publicly available *E. grandis *expressed sequences, and will provide substantial support for the genome sequencing and annotation that are currently under way [11]. Longer reads from the GS-FLX were essential to assemble a reasonable proportion of biologically relevant contig sequences. Although our sequencing effort produced three times more sequence (≈ 148 Mbp) than the control dideoxy-based EST library (Sanger, ≈ 45 Mbp), the 454 short read-based contigs average only one third of the size. Despite this limitation, a substantial gain occurs at the level of gene discovery – the large number of reads in our 454 dataset leads to sampling of sequences from a much broader variety of genes. Therefore, 454-based gene discovery projects represent a viable and, perhaps favorable alternative to Sanger-based sequencing of EST libraries when a diverse sampling of genes is more important than obtaining transcript-length contigs. As GS-FLX becomes the standard 454 pyrosequencing platform, large-scale EST sequencing and gene discovery projects will be more successful in assembly of transcript-length contigs.

We also demonstrated that the detection of valid SNPs is possible by sequencing a pooled sample of highly heterozygous genotypes. By aligning the reads derived from cDNA of 21 *E. grandis *genotypes, we were able to detect 23,742 SNPs and validate 83% of a sample of 337. Therefore, approximately 4,000 of the detected SNPs may be false positives, possibly arising from sequencing errors or alignment of paralogs. Paralogs that share high levels of sequence similarity may have been assembled in the same contig because they cannot be distinguished due to the short read length of 454 pyrosequencing. This would lead to the detection of false SNPs. On the other hand, a higher stringency of assembly raises the possibility of the opposite problem: the separate assembly of haplotypes from highly polymorphic genes. However, the assessment of the EST assembly quality in this study is difficult due to the lack of a reference genome sequence. Nonetheless, the validation rate observed here was similar to the 85% reported for maize, where SNPs were detected by comparing sequences from two separate 454 runs, each interrogating a different inbred homozygous line [7].

A significant number of polymorphisms in our sample may have been overlooked because of the stringent methodology used to declare SNPs. For contigs with reasonable sequence coverage (length > 200 bp) and depth (> 10 reads on average per nucleotide) we detected one SNP for every 192 bp on average. This rate of SNP discovery appears low compared to previous reports of one SNP every ≈ 100 bp in coding sequences of *Eucalyptus *[18,19] and other forest species [20-25]. There are at least three foreseeable reasons for missing true SNPs that are intrinsic to our experimental methodology and SNP detection approach. First, because 454 was used to randomly sequence cDNAs, not all genotypes were sequenced for every SNP locus – in fact, the average sequencing depth (6.7 reads/bp) in our experiment is far lower than the number of possible haplotypes in our sample. Secondly, the requirement that a SNP be observed in at least 10% of the reads aligned to a polymorphic site compromised the sensitivity required to detect rare alleles. Studies have demonstrated an excess of rare alleles in natural populations of forest tree species [22-24] which are probably largely discarded by our approach. Additionally, there is a relatively high level of relatedness among the 21 individuals utilized in our study. The sampled genotypes come from seven open-pollinated families – i.e. seven groups of three individuals share one common maternal parent, limiting the genetic diversity sampled relative to what might be present in a similarly sized sample of unrelated trees. Finally, the detection of polymorphisms was likely hindered by the requirement that at least two reads containing the variant alleles have at least 20 nucleotides of conserved sequence upstream and downstream of the locus. This requirement is intended to minimize the discovery of false polymorphisms due to the alignment of paralogs – a potentially significant problem when aligning short sequence reads. Therefore, only nucleotide variants in relatively conserved or recently derived paralogs may have been incorrectly identified as SNPs. The drawback is that true SNPs in hotspots of genetic diversity or genes under high diversifying selection (e.g. disease-resistance genes, [26]) may be discarded. Considering the high diversity found in forest trees [20-25], the requirement for sequence conservation in regions surrounding a SNP may be too conservative.

High throughput DNA sequencing and SNP discovery from a pool of multiple genotypes may be a powerful approach for rapid assessment of genetic diversity and selection in a genomic scale. Genome-wide surveys of genetic diversity have only recently been reported in the model plant *Arabidopsis *[27]. Here we attempted to generate an approximate estimate of genetic diversity for a broad sample of genes by adapting existing parameters of genetic diversity to our experimental methodology. The most commonly used measure of genetic diversity, the nucleotide diversity parameter theta (θ), is an estimate of the number of polymorphic sites in a random haplotype sample from a population and is independent of the allele frequencies [17,28]. In our study, the number of independent haplotypes sampled is unknown, as the same haplotype may be sampled repeatedly. Therefore we developed a modified nucleotide diversity estimator beta (β) based on SNPs detected by randomly sequencing a multi-genotype cDNA pool. β is always underestimated relative to θ, because the number of reads at a given SNP position is always equal or smaller than the effective (true) number of genotypes. The lower sensitivity to detect SNPs discussed previously also contributes to the underestimation of β. Therefore, the average β reported here (0.00186) is, as expected, much lower than the average θ estimated for genes of *Populus *by 9× [23], Douglas fir by 3.8× [24], loblolly pine by 2.2–2.7× [20,21,29] and Norway Spruce by 1.7× [22]. Although it is not possible to compare estimates of β to the commonly used θ, they are useful for a comparison of nucleotide diversity among genes in this project.

In addition to the nucleotide diversity (β) we also estimated the proportion of non-synonymous to synonymous substitutions (Ka/Ks). As observed in other plant species [13,16,30] most genes of *E. grandis *appear to be under purifying selection and, accordingly, Ka/Ks distribution averages 0.30 and is heavily right-skewed. Among genes predicted to be under strong purifying selection based on the Ka/Ks ratio, there is enrichment for GO categories involving essential biological processes conserved across kingdoms, such as translation, ubiquitin-dependent protein degradation and nucleosome assembly by histones. rRNA and ribosomal proteins have been shown to be highly conserved between species and to evolve under strong purifying selection [31-33]. Several studies also confirm that histone genes evolve constrained by negative selection [34-36].

Among genes classified as diverse and/or under positive selection based on β_N _and Ka/Ks distributions, there is enrichment for genes classified as unknown biological process, defense response and response to biotic stress, and multicellular development. Other researchers have already reported an excess of unknown function among genes under positive selection [16,30,37]. A possible explanation for this overrepresentation is that the category of uncharacterized genes may be enriched for duplicated genes where relaxed selection constraints are leading their diversification and/or eventual silencing [38]. In fact, the category may include pseudogenes and transcribed but untranslated loci. It is also possible that duplicated genes might have higher nucleotide diversity due to the assembly of paralogs. However we do not anticipate false SNPs to bias Ka/Ks estimates because they are not expected to occur more or less frequently in synonymous versus non-synonymous sites by chance.

Genes acting in defense response/response to biotic stimulus are frequently positively selected for diversification to compete with rapidly evolving avirulence genes of pathogens [26,39]. We did find extensive diversity in non-synonymous sites (β_N_) of most defense response genes, but the diversity was also high among synonymous sites and, as a result, these genes were not enriched among those under diversifying selection (measured by the Ka/Ks). Similar results were reported in another study [16]. One possible explanation is that positive selection generally only operates in certain domains (i.e., leucine-rich repeat (LRR)) of resistance genes [26,39] and we estimated Ka/Ks over the entire coding sequence. The multicellular development GO class which is enriched among genes under diversifying selection is mainly due to the presence of NAC transcription factor genes. Transcription factors have been demonstrated to have an excess of positively selected genes in humans [40], and specifically one member of the NAC family was shown to be evolving rapidly in *Arabidopsis *[30]. Finally, the literature survey supports our results and demonstrates that the proposed nucleotide diversity estimator (β) accurately depicts relative differences of variability within gene sequences.

## Conclusion

The unigene sequences being released from this study will provide a much needed public resource for *Eucalyptus *research and will be important for the annotation of the forthcoming *E. grandis *genome sequence. 454-based EST sequencing and *de novo *assembly can provide a foundational transcriptome resource when limited prior sequence information is available. Lastly, we showed that nucleotide diversity of an organism can be sampled by high-throughput sequencing a pool of genotypes, and that this strategy is useful for detecting evolutionary signatures in a large number of genes that are in agreement with smaller scale traditional sequencing projects.

## Methods

### Plant material and RNA extraction

Three greenhouse-grown *Eucalyptus grandis *seedlings from each of seven open-pollinated families (21 genotypes in total) were dissected into xylem (Xy), phloem (Ph), roots (R), young leaves(YL), mature leaves (ML) and apical/lateral meristems (M). Tissues were immediately frozen in liquid nitrogen and stored. For each tissue, RNA was extracted by a standard protocol [41] from a pool of equal proportion of plant material from all 21 genotypes[41,41]. RNA concentration was estimated using an ND-1000 Spectrophotometer (NanoDrop USA, Wilmington, DE) and integrity was evaluated on an agarose gel stained with ethidium bromide.

### cDNA synthesis and normalization

RNA isolated from each tissue pool was combined in varying proportions (10% YL, 10% ML, 15% R, 15% Ph, 20% M and 30% Xy) to a single pool in an attempt to maximize the diversity of transcriptional units sampled. Pooled RNA was DNase treated and purified using the RNAeasy Plant Mini Kit (Qiagen USA, Valencia, CA). Full-length cDNA was synthesized from 2 μg of RNA using the Clontech SMART cDNA Library Construction Kit (Clontech USA, Mountain View, CA) according to manufacturer's protocol, except that the Clontech CDSIII/3' PCR primer was replaced with the Evrogen CDS-3M adaptor (Evrogen, Moscow). cDNA was amplified using PCR Advantage II Polymerase (Clontech USA, Mountain View, CA) in 16 thermo cycles (7s at 95°C, 20s at 66°C, and 4 mins at 72°C) and was subsequently purified using the QIAquick PCR Purification Kit (Qiagen USA, Valencia, CA). The cDNA was normalized using the Evrogen Trimmer-Direct Kit (Evrogen, Moscow) to minimize differences in representation of transcripts. This normalization protocol is based on denaturing-reassociation of cDNAs, followed by digestion with a duplex-specific nuclease (DSN). The enzymatic degradation occurs primarily on the highly-abundant cDNA fraction. The single-stranded cDNA fraction was then amplified twice by sequential PCR reactions according to the manufacturer's protocol. Adaptors incorporated during the first strand synthesis were partially removed by *SfiI *digestion (8 U/μg of cDNA). Normalized cDNA was purified using the QIAquick PCR Purification Kit (Qiagen USA, Valencia, CA).

### 454 sequencing and assembly

Approximately 15 μg of normalized cDNA were used for library construction and sequencing at the Interdisciplinary Center for Biotechnology Research (ICBR) at the University of Florida, following the procedures described by Margulies et al. [1]. Two sequencing runs were produced on the GS-20 platform, and one on the GS-FLX. Bases were called with 454 software by processing the pyroluminescence intensity for each bead-containing well in each nucleotide incorporation. An initial assembly of the sequences was performed with Newbler version 1.1.02.15 (454 Life Science, Branford, CT). Newbler considers the normalized intensity of each nucleotide flow, instead of individual base calls, which is more suited to assembly with sequencing by synthesis like 454. However, Newbler v.1.1.02.15 does not mask sequence repeats like the adaptors used for the cDNA normalization. Thus, after initial assembly of all reads, except those containing adaptor sequences, in Newbler further assembly was performed with Paracel Transcript Assembler (PTA) version 3.0.0 (Paracel Inc., Pasadena, CA). All contigs and singletons resulting from the Newbler assembly were combined with adaptor-containing reads for input into PTA. Using a threshold of 15, all sequences were masked for the presence of oligonucleotide adaptors used during cDNA library preparation and normalization. Low base-call quality (score ≤ 10) data was trimmed from the ends of individual sequences. Low complexity sequence regions (simple sequence repeats) are identified and excluded from consideration during initial pair-wise comparison, but are included during final alignment and consensus building. Assembly is performed in two stages. The first stage uses "Haste" algorithm to build groups (or clusters) of sequences sharing a minimal amount of identifiable sequence similarity (threshold = 50). The second stage carefully assembles sequences within individual clusters into consensus transcripts using the software defaults, except parameters MinCovRep (500), InOverhang (30), EndOverhang (30), RemOverhang (30), QualSumLim (300), MaxInternalGaps (15) and PenalizeN (0). Files containing reads' sequence and quality scores were deposited in the Short Read Archive of the National Center for Biotechnology Information (NCBI) [accession number SRA001122]. Newbler and PTA assembly files were also submitted to NCBI.

### SNP detection and validation

To detect SNPs in the cDNA pool, we used the consensus assembly generated from all sequencing runs as a reference sequence to which individual reads were aligned using GS Reference Mapper (454 Life Science, Branford, CT). Each read was aligned to only a single best homologous site in the reference sequence. Reads aligning equally well in more than one location in the reference were discarded. GS Reference Mapper only scores polymorphisms where two or more reads contain the variant allele. Additionally, at least two reads with the alternative allele must include ≥ 20 bases upstream and downstream of the variable nucleotide and no more than one additional sequence polymorphism in this window. For the analysis reported here, we considered only single nucleotide polymorphisms (SNPs), excluding all indels and variants involving more than one nucleotide. We also imposed the constraint that the variant allele appears in at least 10% of the total number of reads covering the polymorphic site.

To validate a sample of detected SNPs, we designed primers to amplify 500–700 bp of transcripts containing a large number of putative SNPs. Using the normalized cDNA as template, fragments were amplified using PCR with 30 thermocycles of 94°C for 30s, 55°C for 30s and 72°C for 35s. The amplified fragments (amplicons) were purified using the QIAquick PCR Purification Kit (Qiagen USA, Valencia, CA). Each amplicon was sequenced with both forward and reverse primers using standard dideoxy-based technology analyzed on the ABI3730 platform (Applied Biosystems). Sequencing chromatograms were visually analyzed with Chromas 2.32 (Technelysium Pty. Ltd.), and SNPs were identified as overlapping nucleotide peaks. For the SNP to be verified, the variant allele must have a chromatogram peak at least 50% higher than background peaks.

### Analysis of synonymous and nonsynonymous mutations

Protein coding sequence for each consensus was delimited using the best BlastX hit (*E *value 10^-5^) against the *Arabidopsis thaliana *peptides translated from TAIR7 gene models. Codons for each consensus were identified and nucleotide degeneracy determined. The number of synonymous and non-synonymous sites was calculated for each contig. Next, we determined whether SNPs positioned in consensus coding sequences introduce synonymous or nonsynonymous mutations by comparing the translated amino acids from the reference and variant sequences. We calculated the proportion of nonsynonymous to synonymous mutation (Ka/Ks) for each consensus following Hartl and Clark [28], with the exception that one unit was added to both number of synonymous and non-synonymous substitutions. This was important to allow Ka/Ks estimation in cases where either type of substitution was not found. Had we not included this modification, genes with an excess of synonymous but no observed non-synonymous substitutions would all have Ka/Ks equal zero, regardless of their Ks value. On the other hand, genes without any observed synonymous substitution would have undefined Ka/Ks because of division by zero.

## Authors' contributions

EN and DRD synthesized and normalized the cDNA library, summarized sequencing results, annotated the sequences and wrote the manuscript. EN developed the modified methods to estimate nucleotide diversity parameters from 454 sequences. WGF generated and assembled the 454 sequences and identified the SNPs. GJPJ and DG provided the dideoxy-based sequences and did the comparative analysis to the 454 data. RS and MK coordinated and supervised the experiment implementation, and assisted in the analysis of data and manuscript preparation.

## Supplementary Material

Additional file 1 Annotation and Ka/Ks estimates for 2,001 contigs. Annotation and SNP information for the 2,001 *E. grandis *contigs for which Ka/Ks was estimated. Click here for file

Additional file 2 Annotation and β estimates for 2,392 contigs. Annotation of 2,392 contigs for which β was estimated. Provided for each contig are the sequence length, number of non- and synonymous nucleotides sites, average sequencing depth (reads/nt), number of SNPs in non-coding (nc), non-synonymous (ns) and synonymous (s) sites. Click here for file

## References

[B1] Margulies M, Egholm M, Altman WE, Attiya S, Bader JS, Bemben LA, Berka J, Braverman MS, Chen YJ, Chen Z, Dewell SB, Du L, Fierro JM, Gomes XV, Godwin BC, He W, Helgesen S, Ho CH, Irzyk GP, Jando SC, Alenquer ML, Jarvie TP, Jirage KB, Kim JB, Knight JR, Lanza JR, Leamon JH, Lefkowitz SM, Lei M, Li J, Lohman KL, Lu H, Makhijani VB, McDade KE, McKenna MP, Myers EW, Nickerson E, Nobile JR, Plant R, Puc BP, Ronan MT, Roth GT, Sarkis GJ, Simons JF, Simpson JW, Srinivasan M, Tartaro KR, Tomasz A, Vogt KA, Volkmer GA, Wang SH, Wang Y, Weiner MP, Yu P, Begley RF, Rothberg JM (2005). Genome sequencing in microfabricated high-density picolitre reactors. Nature.

[B2] Emrich SJ, Barbazuk WB, Li L, Schnable PS (2007). Gene discovery and annotation using LCM-454 transcriptome sequencing. Genome Res.

[B3] Ohtsu K, Smith MB, Emrich SJ, Borsuk LA, Zhou R, Chen T, Zhang X, Timmermans MC, Beck J, Buckner B, Janick-Buckner D, Nettleton D, Scanlon MJ, Schnable PS (2007). Global gene expression analysis of the shoot apical meristem of maize (Zea mays L.). Plant J.

[B4] Cheung F, Haas BJ, Goldberg SM, May GD, Xiao Y, Town CD (2006). Sequencing Medicago truncatula expressed sequenced tags using 454 Life Sciences technology. BMC Genomics.

[B5] Jones-Rhoades MW, Borevitz JO, Preuss D (2007). Genome-wide expression profiling of the Arabidopsis female gametophyte identifies families of small, secreted proteins. PLoS Genet.

[B6] Weber AP, Weber KL, Carr K, Wilkerson C, Ohlrogge JB (2007). Sampling the Arabidopsis transcriptome with massively parallel pyrosequencing. Plant Physiol.

[B7] Barbazuk WB, Emrich SJ, Chen HD, Li L, Schnable PS (2007). SNP discovery via 454 transcriptome sequencing. Plant J.

[B8] Meyer M, Stenzel U, Myles S, Prufer K, Hofreiter M (2007). Targeted high-throughput sequencing of tagged nucleic acid samples. Nucleic Acids Res.

[B9] Parameswaran P, Jalili R, Tao L, Shokralla S, Gharizadeh B, Ronaghi M, Fire AZ (2007). A pyrosequencing-tailored nucleotide barcode design unveils opportunities for large-scale sample multiplexing. Nucleic Acids Res.

[B10] FAO (2000). Global forest resources assessment 2000 - Main report. FAO Forestry paper 140.

[B11] DOE Joint Genome Institute Announces 2008 Genome Sequencing Targets.. http://www.jgi.doe.gov/News/news_6_8_07.html.

[B12] The Arabidopsis Genome Initiative (2000). Analysis of the genome sequence of the flowering plant Arabidopsis thaliana. Nature.

[B13] Tuskan GA, DiFazio S, Jansson S, Bohlmann J, Grigoriev I, Hellsten U, Putnam N, Ralph S, Rombauts S, Salamov A, Schein J, Sterck L, Aerts A, Bhalerao RR, Bhalerao RP, Blaudez D, Boerjan W, Brun A, Brunner A, Busov V, Campbell M, Carlson J, Chalot M, Chapman J, Chen GL, Cooper D, Coutinho PM, Couturier J, Covert S, Cronk Q, Cunningham R, Davis J, Degroeve S, Dejardin A, Depamphilis C, Detter J, Dirks B, Dubchak I, Duplessis S, Ehlting J, Ellis B, Gendler K, Goodstein D, Gribskov M, Grimwood J, Groover A, Gunter L, Hamberger B, Heinze B, Helariutta Y, Henrissat B, Holligan D, Holt R, Huang W, Islam-Faridi N, Jones S, Jones-Rhoades M, Jorgensen R, Joshi C, Kangasjarvi J, Karlsson J, Kelleher C, Kirkpatrick R, Kirst M, Kohler A, Kalluri U, Larimer F, Leebens-Mack J, Leple JC, Locascio P, Lou Y, Lucas S, Martin F, Montanini B, Napoli C, Nelson DR, Nelson C, Nieminen K, Nilsson O, Pereda V, Peter G, Philippe R, Pilate G, Poliakov A, Razumovskaya J, Richardson P, Rinaldi C, Ritland K, Rouze P, Ryaboy D, Schmutz J, Schrader J, Segerman B, Shin H, Siddiqui A, Sterky F, Terry A, Tsai CJ, Uberbacher E, Unneberg P, Vahala J, Wall K, Wessler S, Yang G, Yin T, Douglas C, Marra M, Sandberg G, de Peer YV, Rokhsar D (2006). The genome of black cottonwood, Populus trichocarpa (Torr. & Gray). Science.

[B14] Moore MJ, Bell CD, Soltis PS, Soltis DE (2007). Using plastid genome-scale data to resolve enigmatic relationships among basal angiosperms. Proc Natl Acad Sci U S A.

[B15] Nei M (2005). Selectionism and neutralism in molecular evolution. Mol Biol Evol.

[B16] Roth C, Liberles DA (2006). A systematic search for positive selection in higher plants (Embryophytes). BMC Plant Biol.

[B17] Watterson GA (1975). On the number of segregating sites in genetical models without recombination. Theor Popul Biol.

[B18] Kirst M, Marques CM, Sederoff RR (2005). Nucleotide diversity and linkage disequilibrium in three Eucalyptus globulus genes.: Pretoria, South Africa..

[B19] Santos SN (2005). Genes de lignificação em Eucalyptus: estrutura e diversidade genética dos genes 4cl e ccoaomt.. Programa de Graduação em Ciências Genômicas.

[B20] Brown GR, Gill GP, Kuntz RJ, Langley CH, Neale DB (2004). Nucleotide diversity and linkage disequilibrium in loblolly pine. Proc Natl Acad Sci USA.

[B21] Gonzalez-Martinez SC, Ersoz E, Brown GR, Wheeler NC, Neale DB (2006). DNA sequence variation and selection of tag SNPs at candidate genes for drought-stress response in Pinus taeda L. Genetics.

[B22] Heuertz M, De Paoli E, Kallman T, Larsson H, Jurman I, Morgante M, Lascoux M, Gyllenstrand N (2006). Multilocus patterns of nucleotide diversity, linkage disequilibrium and demographic history of Norway spruce [Picea abies (L.) Karst]. Genetics.

[B23] Ingvarsson PK (2005). Nucleotide polymorphism and linkage disequilbrium within and among natural populations of European Aspen (Populus tremula L., Salicaceae). Genetics.

[B24] Krutovsky KV, Neale DB (2005). Nucleotide diversity and linkage disequilibrium in cold-hardiness- and wood quality-related candidate genes in Douglas fir. Genetics.

[B25] Ma XF, Szmidt AE, Wang XR (2006). Genetic structure and evolutionary history of a diploid hybrid pine Pinus densata inferred from the nucleotide variation at seven gene loci. Mol Biol Evol.

[B26] Bergelson J, Kreitman M, Stahl EA, Tian D (2001). Evolutionary dynamics of plant R-genes. Science.

[B27] Clark RM, Schweikert G, Toomajian C, Ossowski S, Zeller G, Shinn P, Warthmann N, Hu TT, Fu G, Hinds DA, Chen H, Frazer KA, Huson DH, Scholkopf B, Nordborg M, Ratsch G, Ecker JR, Weigel D (2007). Common sequence polymorphisms shaping genetic diversity in Arabidopsis thaliana. Science.

[B28] Hartl DL, Clark AG (2007). Molecular population genetics.. Principles of population genetics.

[B29] Neale DB, Savolainen O (2004). Association genetics of complex traits in conifers. Trends Plant Sci.

[B30] Barrier M, Bustamante CD, Yu J, Purugganan MD (2003). Selection on rapidly evolving proteins in the Arabidopsis genome. Genetics.

[B31] McIntosh KB, Bonham-Smith PC (2001). Establishment of Arabidopsis thaliana ribosomal protein RPL23A-1 as a functional homologue of Saccharomyces cerevisiae ribosomal protein L25. Plant Mol Biol.

[B32] Rooney AP, Ward TJ (2005). Evolution of a large ribosomal RNA multigene family in filamentous fungi: birth and death of a concerted evolution paradigm. Proc Natl Acad Sci U S A.

[B33] Stage DE, Eickbush TH (2007). Sequence variation within the rRNA gene loci of 12 Drosophila species. Genome Res.

[B34] Eirin-Lopez JM, Gonzalez-Tizon AM, Martinez A, Mendez J (2004). Birth-and-death evolution with strong purifying selection in the histone H1 multigene family and the origin of orphon H1 genes. Mol Biol Evol.

[B35] Matsuo Y, Yamazaki T (1989). Nucleotide variation and divergence in the histone multigene family in Drosophila melanogaster. Genetics.

[B36] Rooney AP, Piontkivska H, Nei M (2002). Molecular evolution of the nontandemly repeated genes of the histone 3 multigene family. Mol Biol Evol.

[B37] Cork JM, Purugganan MD (2005). High-diversity genes in the Arabidopsis genome. Genetics.

[B38] Lynch M, Conery JS (2000). The evolutionary fate and consequences of duplicate genes. Science.

[B39] Fluhr R (2001). Sentinels of disease. Plant resistance genes. Plant Physiol.

[B40] Bustamante CD, Fledel-Alon A, Williamson S, Nielsen R, Hubisz MT, Glanowski S, Tanenbaum DM, White TJ, Sninsky JJ, Hernandez RD, Civello D, Adams MD, Cargill M, Clark AG (2005). Natural selection on protein-coding genes in the human genome. Nature.

[B41] Chang S, Puryear J, Cairney J (1993). A simple and efficient method for isolating RNA from pine trees. Plant Mol Biol Rep.

